# Changes in cultured endothelial cell glycosaminoglycans under hyperglycemic conditions and the effect of insulin and heparin

**DOI:** 10.1186/1475-2840-8-46

**Published:** 2009-08-20

**Authors:** Juying Han, Fuming Zhang, Jin Xie, Robert J Linhardt, Linda M Hiebert

**Affiliations:** 1 Department of Veterinary Biomedical Sciences, University of Saskatchewan, Saskatoon, Saskatchewan S7N 5B4, Canada; 2 Departments of Chemistry and Chemical Biology, Biology, and Chemical and Biological Engineering, Center for Biotechnology and Interdisciplinary Studies, Rensselaer Polytechnic Institute, Troy, NY 12180, USA

## Abstract

**Background:**

Heparan sulfate proteoglycans (HSPGs) contain glycosaminoglycan (GAG) chains made primarily of heparan sulfate (HS). Hyperglycemia in diabetes leads to endothelial injury and nephropathy, retinopathy and atherosclerosis. Decreased HSPG may contribute to diabetic endothelial injury. Decreased tissue HS in diabetes has been reported, however, endothelial HS changes are poorly studied.

**Objective:**

To determine total GAGs, including HS, in endothelium under hyperglycemic conditions and the protective effect of insulin and heparin.

**Methods:**

Confluent primary porcine aortic endothelial cells (PAECs) were divided into control, glucose (30 mM), insulin (0.01 unit/ml) and glucose plus insulin treatment groups for 24, 48 and 72 hours. Additionally, PAECs were treated with glucose, heparin (0.5 *μ*g/ml) and glucose plus heparin for 72 hours. GAGs were isolated from cells and medium. GAG concentrations were determined by the carbazole assay and agarose gel electrophoresis.

**Results:**

GAGs were significantly increased only in control and glucose plus insulin groups at 72 versus 24 hours. Glucose decreased cell GAGs and increased medium GAGs, and insulin alone decreased cell GAGs at all times compared to control. In the glucose plus insulin group, cell GAGs were less than control at 24 hours, and greater than glucose or insulin alone at 48 and 72 hours while GAGs in medium were greater than control at all times and glucose at 72 hours. Heparin increased GAGs in glucose treated cells and medium.

**Conclusion:**

High glucose and insulin alone reduces endothelial GAGs. In hyperglycemic conditions, heparin or insulin preserves GAGs which may protect cells from injury. Insulin is an effective diabetic therapy since it not only lowers blood glucose, but also protects endothelium.

## Background

A glycosaminoglycan (GAG) is a linear, highly negatively charged, polysaccharide macromolecule possessing a disaccharide repeat sequence which usually comprises an amino sugar with D-glucosamine or galactosamine and a uronic acid residue of either D-glucuronic acid or iduronic acid. Based on disaccharide composition, GAG structures are categorized into six common types including chondrotin sulfate (CS), dermatan sulfate (DS), heparan sulfate (HS), keratan sulfate (KS), hyaluronic acid, and heparin. The GAG chain is covalently attached to a core protein through an O-link to serine or an N-link to an asparagine residue and this structure is called a proteoglycan. Proteoglycans are expressed by all mammalian cells and are found on the cell surface, in the extracellular matrix (ECM) and intracellular granules [1].

HS is a prominent blood vessel component and the most common GAG found on the endothelial cell (EC) surface and in the ECM. Heparan sulfate proteoglycans (HSPGs) are receptors for circulating growth factors and chemokines that regulate cell proliferation, differentiation and migration [2]. The interaction of HS side chains with bioactive factors controls physiological processes of embryonic development, tissue repair, blood coagulation, cartilage function and glomerular filtration [1,3,4]; and pathological processes of wound healing, vessel formation, and tumor cell growth, adhesion, invasiveness and metastasis [5]. HS degradation is considered a major cause of endothelial dysfunction resulting in disturbance of vascular integrity and barrier properties, due to decreased negative charge and increased permeability, and release of bioactive substances such as cytokines, enzymes and growth factors bFGF and TGF-*β*. Changes in proteoglycan expression, as well as structural and functional alterations of their GAG components, are associated with cardiovascular disease, cancer, inflammation, amyloidosis and diabetes [6-9].

Several studies suggest that HS degradation is associated with diabetic nephropathy [10-12]. An increased glomerular albumin filtration rate was observed in microalbuminuric patients with overt diabetes [13]. Increased glomerular basement membrane (GBM) permeability was associated with diminished GBM HS content [10]. Similar changes in HS content was observed in aortic intimas of diabetic patients [14]. HS in skin basement membrane in patients with diabetic nephropathy was also significantly reduced compared to those without nephropathy. Similarly, reduced ^35^S-labeled HSPG synthesis was observed in aorta, liver and intestinal epithelium of diabetic rats [15-17]. This evidence suggests that changes in HS metabolism occur not only in the kidney but in any tissue or organ in the diabetic condition, indicating a link between HS abnormalities and vascular complications both in the micro and macro vasculature. In addition, heparanase, an endoglucuronidase produced by ECs induced by high glucose, could be responsible for the cleavage of HS chains [18]. Heparanase activity was increased in the urine of diabetic patients [19] and reduction of HS moieties in the glomeruli of patients with overt diabetic nephropathy in type II diabetes was correlated with heparanase upregulation [11]. However, specific changes in GAGs from ECs under hyperglycemic conditions are not well studied. The purpose of this study was to use primary porcine aortic ECs (PAECs) under high glucose conditions as an in vitro model to determine if changes in GAGs are seen with hyperglycemia. Since the hormone insulin regulates glucose metabolism and promotes glucose uptake and utilization to reduce glucose concentration in hyperglycemia, we also wished to determine the effect of insulin on GAG alterations. Since our previous studies showed that heparin also protected ECs from high glucose injury [20], the effect of heparin was also studied.

## Methods

### Porcine Aortic Endothelial Cell (PAEC) Culture

Primary PAECs were cultured by the method of Gotlieb and Spector [21]. Porcine aortic segments obtained from local abattoirs were washed three times in Ca^2+^-Mg^2+^-free Dulbecco's phosphate-buffered saline (CMF-DPBS) and connective tissue was trimmed from the adventitial surface. The aorta was held upright by clamping one end with hemostats. The aortic lumen was rinsed three times with CMF-DPBS and then filled with collagenase (Type IV, SIGMA, St. Louis, MO, USA; 1 mg/ml in CMF-DPBS) for six minutes. After removing the collagenase, the lumen was gently rinsed with medium (M199 containing 5.5 mM D-glucose, without heparin or insulin, GibcoBRL, Life Technologies, Inc., Grand Island, NY, USA) supplemented with 5% fetal bovine serum (FBS, GibcoBRL), 50 *μ*g/ml penicillin (SIGMA) and 10 *μ*g/ml streptomycin (SIGMA). The medium with cells was plated onto 60 mm culture dishes which were then incubated at 37°C with 5% CO_2_/95% air in a humidified environment. PAECs were identified by their morphological appearance of cobblestone-like flattened cells, and the presence of von Willebrand factor (vWF) in initial cultures. Non-endothelial-like cells, such as smooth muscle cells and fibroblasts were destroyed cell by cell using a pasteur pipette and mechanical suction before the first passage. To pass cells, confluent cultures were washed twice with CMF-DPBS and cells were detached by trypsin (0.025% with EDTA in CMF-DPBS) for two or three minutes at room temperature. The cells were resuspended in medium and transferred to 60 mm dishes for further passage. For experiments, the cells were grown in 60 or 100 mm dishes at passage four.

### Treatment of Cultures

PAECs were grown in 100 mm culture dishes (surface area 78.5 cm^2^/dish) with an estimated 6 million cells/dish at confluence. Cells were incubated with four different treatments (3 dishes/group) including control (serum free medium), glucose (30 mM), insulin (0.01 unit/ml), or glucose plus insulin in serum-free medium for 24, 48 and 72 hours. In a separate experiment ECs in 100 mm dishes were treated with high glucose (30 mM) and/or heparin (0.5 *μ*g/ml), or serum free medium (control) with one dish/group for 72 hours. Additionally, PAECs were grown in 60 mm dishes and treated as controls or with high glucose (30 mM) for 24, 48 and 72 hours with 3 dishes/group. After the specified time interval, cell medium was collected into 15 ml centrifuge tubes. Cells were scraped from the dish using a cell scraper, the dish was washed with DPBS, and cells and washings were collected into a 1.5 ml tube and centrifuged to pellet cells. The cell pellets and medium were frozen at -80°C. Samples were freeze-dried before they were analyzed.

### Extraction of Total GAGs

Medium and cell pellets were digested by pronase (0.2 mg/ml in 0.1 M Tris buffer containing 0.1 M CaCl_2_, pH 8.0) for 24 hours at 37°C. Then the digested samples were freeze-dried a second time. Total GAG extraction was performed using Vivapure Ion Exchange Mini Spin Columns (Vivascience, Germany). Urea buffer (8 M urea with 2% w/v CHAPS (3-[(3-Cholamidopropyl) dimethylammonio]-1-propanesulfonate), 500 *μ*l, was added to the samples which were then triturated with a pipette to insure homogenization. The samples were then centrifuged at 5000 × g for 5 minutes to remove the precipitate. The supernatant was added into the Spin Columns which had previously been equilibrated with 500 *μ*l urea buffer followed by centrifugation. The columns with supernatant were centrifuged at 5000 × g for 10 minutes to remove the flow-through. Columns were washed once with 500 *μ*l of urea buffer. Then columns were washed five times with 500 *μ*l of 200 mM NaCl, after which total GAGs were eluted with 3 × 100 *μ*l of 16% NaCl. To precipitate the total GAGs, 1.2 ml of methanol was added to the sample, making the final methanol concentration 80% of volume, and chilled overnight at -4°C. The total GAG fraction was collected by centrifugation at 2500 × g for 15 min. The resulting precipitate was dissolved in 100 *μ*l of distilled water.

### Determination of Total GAGs by the Carbazole Assay

The carbazole assay was used to examine the total GAGs isolated from cultured PAECs and medium in experiments where cultures were treated with high glucose and/or insulin. Briefly, 25 *μ*l of the extracted GAG was added to 150 *μ*l of reagent A (25 mM Na_2_B_4_O_7 _in concentrated H_2_SO_4_) which was heated at 100°C for 10 minutes. After the reaction was cooled on ice, 5 *μ*l of reagent B (carbazole 1.25 g in 1 L 100% ethanol) was added to the reaction which was heated at 100°C for another 15 minutes. The absorbance was read by spectrometry at 525 nm using a 96-well micro titer plate. The concentrations of GAGs were calculated by comparing to a standard curve made with heparan sulfate from porcine intestinal mucosa (SIGMA).

### Gel Electrophoresis Analysis of GAGs

Agarose gel electrophoresis described by Jaques [22] was used to identify GAGs recovered from control ECs and ECs treated with glucose, insulin and glucose+insulin for 72 hours, and with glucose, heparin and glucose+heparin for 24 hours. Total GAGs were extracted from cells and medium as describe above. Then, 3–5 μl distilled water was added to each dried GAG sample and 2 μl of the dissolved GAG was added to the lanes on the agarose (1%) gel electrophoresis slide. Slides were run in barbital buffer (pH 8.6) for 20 minutes. Slides were then fixed in 0.01% cetavlon for at least 2 hours. After drying, slides were stained with 0.04% Toluidine Blue in 80% acetone and background colour was removed with 1% acetic acid. Heparan sulfate was identified and visualized by comparing to reference heparan sulfate from porcine intestinal mucosa (0.1 *μ*g/μl, SIGMA).

### Determination of Total HS Disaccharides in Cells and Medium

Additional experiments were performed for determination of HS disaccharides in cells and medium. Total GAGs from three 60 mm dishes were combined in order to obtain enough material for HS disaccharide analysis by HPLC. Freeze-dried total GAG extracts were dissolved in 20 *μ*l of 0.2 M Tris-HCl buffer (pH 8.0), then 2 *μ*l of Chondroitinase ACII (0.05 unit/*μ*l) and 10 *μ*l of Chondroitinase ABC (0.01 unit/*μ*l) were added to the samples which were incubated at 37°C overnight. Microcon YM-3, 3000 NMWL Centrifugal Filter Devices (Millipore, USA) were used to obtain HS disaccharides. First, the device was washed with 0.1 M NaOH once followed by washing with 50 *μ*l H_2_O four to five times until the pH of the flow-through was neutral. Second, the chondroitinase digested samples were transferred to the devices and centrifuged at 10,000 × g for 25 minutes to remove chondroitin sulfates. After the devices were washed with double distilled (DD) H_2_O three to four times, the inserts were removed and put into a new collecting tube. A mixture of 10 *μ*l of heparinase I (0.3 unit), heparinase II (0.1 unit) and heparinase III (0.1 unit) in 6 mM NaCl and 3 mM Na_3_PO_4 _buffer (pH 7.1) were added to the column. After incubation at 37°C for 24 hours to digest HS, the samples were centrifuged at 10,000 × g for 10 minutes to remove the buffer. Then HS disaccharides were eluted by three to four additions of 50 *μ*l DD H_2_O. The elution was freeze-dried and then dissolved in 15 *μ*l DD H_2_O. Then 10 *μ*l of the sample was analyzed by HPLC. The analysis was performed on a post-column fluorescence RPIP-HPLC using Shimazu LC-10Ai system equipped with a RF535 fluorescence HPLC monitor and an analytical C18 column (5 μm, 4.6 × 250 mm). The mobile phase was A: 8% acetonitrile, 3 mM tributylamine acetate, pH 5.0; B: 8% acetonitrile, 3 mM tributylamine acetate, 0.2 M NaCl, pH 5.0 with a step linear gradient 0–15 min, B 0–30%; 15–30 min, B 30–100%; 30–50 min, B 100% at a flow rate of 1.0 ml/min. Column temperature was 55°C. Post-column reaction reagents were C: 1% 2-cyanoacetamite and D: 0.75 M NaOH with a flow rate at 0.2 ml/min. The reaction temperature was 120°C and detected with Ex 346 nm, Em 410 nm.

### Statistical Analysis

Data for total GAGs analysed by the carbazole assay are expressed as mean +/- standard error (SE) from three dishes per group. A one-way ANOVA was used to determine significant differences between groups. Values of *P < 0.05 *were considered to be statistically significant. A one-tailed t-test was used to determine significant differences between total GAGs at 24 and 72 hour.

## Results

### Total GAGs in Cultures

Total GAGs were extracted from cells and medium grown in 100 mm dishes after 24, 48 and 72-hour treatment with glucose (30 mM) and/or insulin (0.01 unit/ml).

#### 24 hours

**Figure 1:**
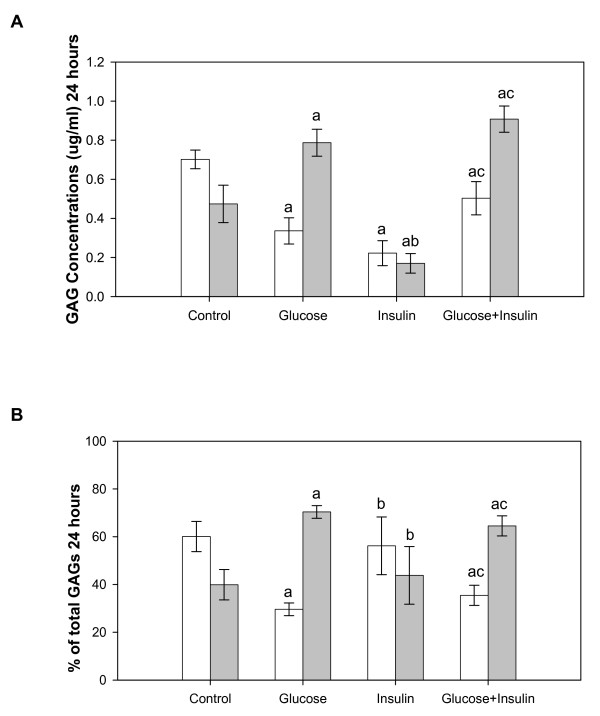
**GAGs from Cultured Endothelial Cells and Medium at 24 Hours**. Cells were treated with high glucose (30 mM) and/or insulin (0.01 unit/ml) for 24 hours. Total GAGs were isolated from cells (white bar) and medium (grey bar) and determined by the carbazole assay. Three dishes/group. Data were analyzed by a one-way ANOVA. Significantly different than: a, Control; b, Glucose; c, Insulin. *p < 0.05*.

The GAG concentration was significantly decreased in high glucose or insulin treated cells compared to control (Figure 1A). When high glucose was present, insulin significantly increased GAG concentrations in cells compared to insulin alone, although concentrations were still significantly less than control cells. In medium from the same cultures, GAG concentrations were significantly increased in high glucose and decreased with insulin treatment compared to control. GAG concentrations in medium were significantly increased by glucose+insulin compared to control and insulin alone treated cultures. When the ratio of GAGs in cells and medium was expressed as a percentage, there were more GAGs in cells than medium in control and insulin alone treated cultures, while in high glucose treated cultures with and without insulin the ratio was reversed (Figure 1B). The percentage of total GAGs in glucose treated cells was significantly lower than control and with insulin alone. The percentage of total GAGs in high glucose treated medium with and without insulin was significantly increased compared to control and insulin alone.

#### 48 hours

**Figure 2:**
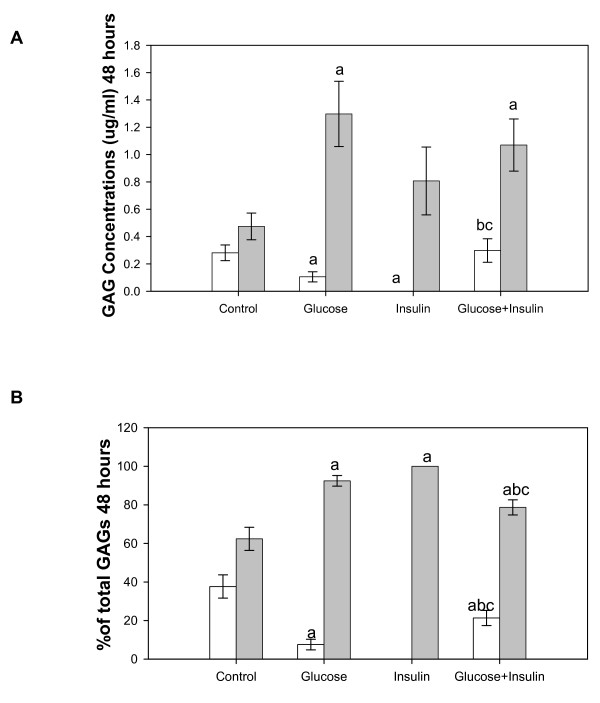
**GAGs from Cultured Endothelial Cells and Medium at 48 Hours**. Cells were treated with high glucose (30 mM) and/or insulin (0.01 unit/ml) for 48 hours. Total GAGs were isolated from cells (white bar) and medium (grey bar) and determined by the carbazole assay. Three dishes/group. Data were analyzed by a one-way ANOVA. Significantly different than: a, Control; b, Glucose; c, Insulin. *p < 0.05*.

GAG concentrations in cells were significantly decreased in high glucose or insulin treated compared to control cultures. Total GAGs in insulin treated cells when high glucose was present was significantly greater than high glucose or insulin alone and similar to control cells (Figure 2A). In the same cultures GAG concentration in medium was significantly increased in high glucose and high glucose plus insulin treated compared to control cultures. When GAG percentages were calculated more GAGs were found in medium than cells for all groups. The percentage of total GAGs in cells was decreased in all treatments compared to controls and increased in insulin plus high glucose compared to high glucose or insulin alone (Figure 2B). The percentages of total GAGs in medium were increased in all treatment groups compared to control and decreased in high glucose plus insulin treated cultures compared to high glucose and insulin alone.

#### 72 hours

**Figure 3:**
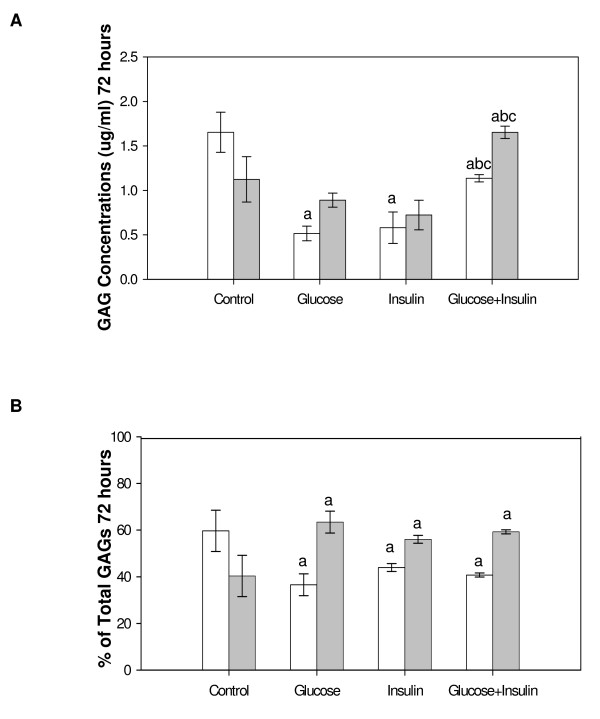
**GAGs from Cultured Endothelial Cells and Medium at 72 Hours**. Cells were treated with high glucose (30 mM) and/or insulin (0.01 unit/ml) for 72 hours. Total GAGs were isolated from cells (white bar) and medium (grey bar) and determined by the carbazole assay. Three dishes/group. Data were analyzed by a one-way ANOVA. Significantly different than: a, Control; b, Glucose; c, Insulin. *p < 0.05*.

Relative GAG concentrations in cells were similar to those seen at 24 hours and significantly less than control for all treatment groups. High glucose+insulin significantly increased GAG concentrations compared to high glucose or insulin alone (Figure 3A). The GAG concentrations in medium were increased in glucose+insulin treated cultures compared to the other three groups. When GAG percentages in cells and medium were calculated more GAGs were found in medium than cells for all experimental groups while in controls the cells contained most of the GAGs. The percentage of total GAGs significantly decreased in cells and increased in medium for all treatments compared to control (Figure 3B).

### Comparison of Total GAGs in Cultures at Different Times

**Figure 4:**
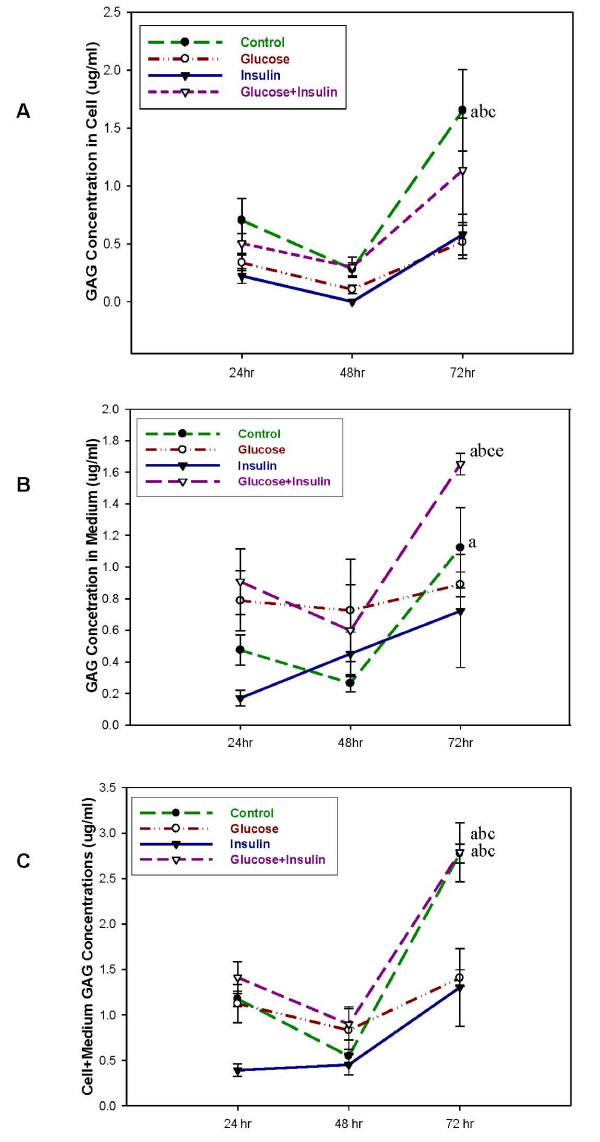
**Total GAGs Increased with Culture Time in Both Cells and Medium**. Cells were treated with high glucose (empty dot, 30 mM), insulin (solid triangle, 0.01 unit/ml), glucose+insulin (empty triangle) and control (solid dot) for 24, 48, and 72 hours (3 dishes/group). Total GAGs isolated from cells (A), medium (B) and cells +medium (C) and determined by the carbazole assay are shown. A one-tailed t-test was used to determine significant differences between treatment at 24 and 72 hour. Significantly greater than: a, same group at 24 hours; b, glucose at 72 hours; c, insulin at 72 hours; d, glucose+insulin at 72 hours; e, control at 72 hours. *p < 0.05*.

GAG concentrations were significantly increased in control cells and in control and glucose+insulin treated medium at 72 compared to 24 hours. GAG concentrations in control cells at 72 hours were greater than high glucose and insulin alone treated cells at 72 hours. GAG concentrations in glucose plus insulin treated medium were greater than all other treatment groups at 72 hours (Figure 4A and 4B). Significantly more GAGs were found in cells plus medium in control and high glucose plus insulin treated cultures at 72 compared to 24 hours and to other groups at 72 hours (Figure 4C).

### GAG Analysis by Gel Electrophoresis

**Figure 5:**
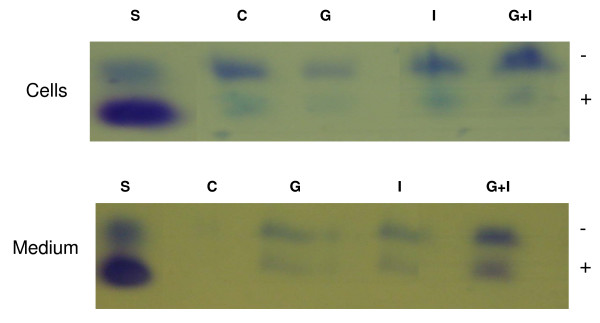
**Gel Electrophoresis Showing GAGs in Cells and Medium in Cultures Treated with Glucose and/or Insulin for 72 Hours**. Lanes: S, standard, heparan sulfate from porcine intestinal mucosa; C, control; G, glucose; I, insulin; G+I, glucose+insulin (3 dishes combined/group)

**Figure 6:**
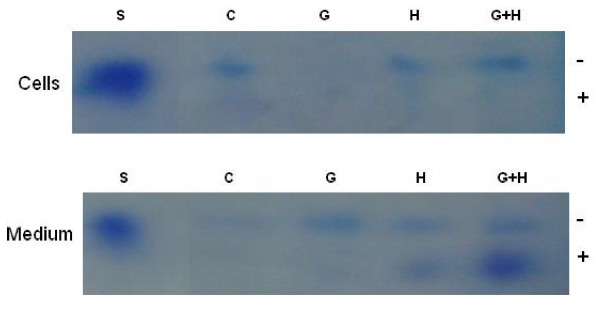
**Gel Electrophoresis Showing GAGs in Cells and Medium Treated with Glucose and/or Heparin for 72 Hours**. Lanes: S, standard, heparan sulfate from porcine intestinal mucosa; C, control; G, glucose; H, heparin; G+H, glucose+heparin (1 dish/group).

In order to better define GAGs extracted from cells treated with high glucose and/or insulin for 72 hours, GAGs were exposed to agarose gel electrophoresis, as shown in Figure 5. In agreement with the carbazole assay at 72 hours, control cells showed a higher GAG content than medium, while with glucose treatment there were less GAGs in cells than medium. Glucose plus insulin treatment showed a greater GAG content than insulin treatment alone both in cells and medium. In insulin alone treated cultures, the faster migrating component was reduced in cells compared to control and high glucose treated cells. Since heparin interferes with HS determination in HPLC and the carbazole assay, GAGs extracted from cells treated with high glucose and/or heparin for 72 hours were only determined and distinguished on gel electrophoresis, as shown in Figure 6. Control and glucose treated cells showed results similar to those mentioned above for glucose and/or insulin treatment. Glucose plus heparin had more GAG content in cells compared to glucose or heparin alone and in medium compared to heparin alone. In medium the fast migrating band was greatest for heparin plus glucose treatment while the slower migrating bands were greater for the treated group than control.

### Correlation of Total HS Disaccharides and Total GAGs

**Figure 7:**
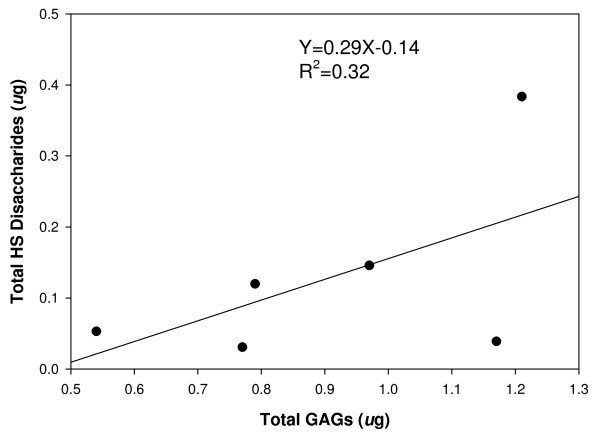
**Correlation of Total HS Disaccharides and Total GAGs in Cultured Endothelial Cells**. Control cells and cells treated with high glucose (30 mM) were cultured for 24, 48 and 72 hours (3 dishes/group). Total GAGs were isolated, after cells and medium separately from the three dishes were determined by the carbazole assay. HS disaccharides were obtained by heparinase digestion and determined by RPIP-HPLC systems.

Control cells and cells treated with high glucose (30 mM) (three 60 mm dishes/group) were cultured for 24, 48 and 72 hours. Total GAGs and HS disaccharides were obtained from the same samples. There was a good correlation between total HS disaccharides and total GAGs obtained (Figure 7). The type of HS disaccharides recovered did not differ between high glucose and control samples (data not shown).

## Discussion

Syndecans and perlecan are major HSPGs synthesized and secreted by ECs. Syndecans are mainly expressed on the cell surface and perlecan is presented in the ECM [23]. Heparan sulfate chains derived from HSPG are the main GAGs found in ECs [24]. In the present study, we found that total HS determined by HPLC correlated well with total GAGs determined by the carbazole assay (Figure 7). Therefore, total GAGs likely reflect total HS present within endothelium. The GAGs were extracted from the entire cultured endothelial monolayer including mainly syndecans and perlecan.

Total GAGs from the cells and medium were determined when cultured ECs were exposed to high glucose and/or insulin. At the earliest observed time (24 hours), cells treated with high glucose showed a reduction in total GAGs (Figure 1). The observation that cell total GAGs decreased and medium GAGs increased up to 72 hours with high glucose treatment (Figure 3, 4, and 5) are consistent with the finding that GAG contents from syndecan-1 and perlecan were decreased in cultured primary ECs treated with high glucose for two and five days [25,26]. Decreased cell GAGs suggest hyperglycemia could induce HSPG degradation or inhibit HSPG synthesis in ECs. The possible consequences of decreased GAGs are reduced interaction of several biofactors such as growth factors, coagulation factors, chemokines, adhesion molecules, and lipoprotein lipase with HSPG in the vasculature [27]. Loss of these biofactors is likely to induce endothelial injury. We previously observed endothelial injury following addition of high glucose in the same culture model [20]. Although our culture model uses aortic ECs, and ECs in macro- and micro-vessels have different properties, both are characterized by the same pathological features in diabetes mellitus. Endothelial injury likely contributes to diabetic cardiovascular complications both in micro- and macro-vessels such as nephropathy, retinopathy and atherosclerosis [28,29].

Increased total GAGs in medium with high glucose treatment suggest that GAGs are released from cell proteoglycan core proteins. The core protein may remain on the cell surface. This was indirectly confirmed by immunoprecipitation assays where the core protein of syndecan-1 remained on ECs and was expressed at lower intensity in medium from high glucose treated ECs [24]. Similarly, immunostaining studies showed that reduction in HS GAGs in the GBM under diabetic conditions was not accompanied by a reduction in the HSPG core protein [30]. As well, decreased HSPG synthesis by human aortic ECs in high glucose conditions was not the result of a decrease in GAG size, further suggesting that entire GAG chains were released into culture medium [25]. It is unlikely that GAGs are further degraded in medium.

Insulin controls blood glucose utilization and influences the metabolism of fat and protein. Insulin also has effects on the expression of numerous genes. Insulin alone decreased GAG concentration in cells at all time points in our study. In a study of regulation of HSPG metabolism and hepatocyte growth by insulin and phosphatidylinositol, insulin markedly stimulated the rate of internalization of matrix HSPG and phospholipase C and therefore may control cell surface HSPG turnover [31]. Thus insulin may regulate enzymes involved in metabolism of proteoglycans. Insulin promoted shedding of syndecan ectodomains, from 3T3-L1 adipocytes, by an unknown mechanism [32]. However, it is unknown whether these changes are coordinated by phosphatidylinositol, the second messenger in the action of binding insulin to its receptor, and whether shedding of syndecans are seen in ECs in response to insulin. In our studies there was a trend that insulin alone increased GAGs in culture medium as culturing time increased (Figure 4) which may indicate HSPG turnover or shedding of syndecan into medium, however, GAGs were significantly reduced in 72 hour cultures compared to control suggesting that GAG synthesis may also be inhibited. These limited observations cannot define the precise mechanisms involved in insulin reduction of GAGs in cultured ECs, and further investigation is required. Considerable evidence indicates that vascular endothelium is a physiological target of insulin and a potential link between insulin resistance and atherosclerosis [33,34]. Endothelial dysfunction is one of the earliest detectable signs in insulin resistance [35]. Our present study shows that decreased GAG content in insulin alone treated cells may lead to endothelial dysfunction caused by GAG degradation and/or inhibition of GAG synthesis.

The evidence for insulin influencing different cultured cells under high glucose conditions is varied and controversial. Studies on cultured mesangial cells treated with glucose and insulin showed insulin did not influence HSPG content independent of the ambient glucose levels [36]. Insulin was unable to correct the 30 mM glucose induced reduction in HSPG synthesis of rat glomerular epithelial cell layers that resemble the GBM [37]. However, reduction in chondroitin sulfate proteoglycan synthesis found in articular cartilage in diabetic rats could be completely restored by administration of insulin [38]. Administration of large doses of insulin restored HSPG synthesis in basement membrane following reduction of HSPG by implantation of Engelbreth-Holm-Swarm (EHS) tumor cells in diabetic mice [39]. Our current studies showed that: insulin increased GAGs in high glucose treated cells compared to insulin alone at all times and to high glucose alone at 48 and 72 hours; insulin increased medium GAGs at all times compared to control, at 24 and 72 hours compared to insulin alone; insulin was able to maintain high glucose treated cell+medium GAGs at control levels at 72 hours (Figure 4C). Previous studies noted that insulin alone increased endothelin-1 (ET-1) levels [40]. Perhaps insulin maintains HSPGs under hyperglycemic conditions through its effects on ET-1. Although these studies indicate that insulin has the potential to improve hyperglycemia induced alteration of HSPG in ECs, the effect is time dependent. It is possible that insulin modulation of proteoglycan metabolism under hyperglycemic conditions is cell or tissue specific and specific for individual species of proteoglycans.

Heparin used as an antithrombotic drug, is also considered a potent vasodilator [41], and lowers blood pressure [42]. Heparin has the ability to inhibit heparanase upregulation induced by high glucose in cultured ECs [18]. Our results suggest that heparin increases GAG content in cells treated with high glucose suggesting the protective effect of heparin on ECs. Taken together with evidence that heparin stimulates HS synthesis and modification of HS in ECs [43,44], further suggests that heparin's protective action may be due to an increase in GAG synthesis or inhibition of heparanase production.

## Conclusion

ECs are targets for hyperglycemia in diabetes mellitus resulting in cardiovascular complications. Alteration of GAGs synthesized by cells is an important pathological mechanism, which can be correlated with cell injury by hyperglycemia. Insulin or heparin undoubtedly protects cell GAGs from degradation and/or increases GAG synthesis which may protect cells from high glucose injury. These results confirm the efficacy of insulin as a therapeutic drug for the diabetic patient since it not only lowers blood glucose levels, but also protects the vasculature. The effectiveness of heparin on the protection of ECs through maintaining GAG content was also confirmed. Elevated insulin alone without hyperglycemia may have a damaging effect. The exact mechanism of insulin influence on EC HSPG metabolism needs to be further elucidated at the levels of gene expression and cell signaling pathways.

## Abbreviations

CMF-DPBS: Ca^2+^-Mg^2+^-free Dulbecco's phosphate-buffered saline; CS: chondrotin sulfate; DD: double distilled; DS: dermatan sulfate; EC: endothelial cell; ECM: extracellular matrix; EHS: Engelbreth-Holm-Swarm; ET-1: endothelin-1; GAG: glycosaminoglycan; GBM: glomerular basement membrane; HS: heparan sulfate; HSPGs: Heparan sulfate proteoglycans; KS: keratan sulfate; PAECs: porcine aortic endothelial cells; SE: standard error; vWF: von Willebrand factor

## Competing interests

The authors declare that they have no competing interests.

## Authors' contributions

JH participated in the study design, performed the experiments, analyzed data, interpreted results and wrote the manuscript; FZ coordinated and participated in the carbazole assay; JX participated in HPLC analysis; RJL coordinated the study and was involved in data interpretation; LMH conceived the study design, participated in data interpretation and critically reviewed the manuscript.

## References

[B1] Kjellen L, Lindahl U (1991). Proteoglycans: Structures and Interactions. Annu Rev Biochem.

[B2] Linhardt RJ, Toida T (1997). Carbohydrates As Drugs.

[B3] Bernfield M, Kokenyesi R, Kato M, Hinkes MT, Spring J, Gallo RL, Lose EJ (1992). Biology of the Syndecans: a Family of Transmembrane Heparan Sulfate Proteoglycans. Annu Rev Cell Biol.

[B4] Salmivirta M, Jalkanen M (1995). Syndecan Family of Cell Surface Proteoglycans: Developmentally Regulated Receptors for Extracellular Effector Molecules. Experientia.

[B5] Vlodavsky I, Bar-Shavit R, Korner G, Fuks Z (1993). Extracellular Matrix Bound Growth Factors, Enzymes and Plasma Proteins.

[B6] Rosenberg RD, Shworak NW, Liu J, Schwartz JJ, Zhang L (1997). Heparan Sulfate Proteoglycans of the Cardiovascular System. Specific Structures Emerge but How Is Synthesis Regulated?. J Clin Invest.

[B7] Iozzo RV, Cohen I (1993). Altered Proteoglycan Gene Expression and the Tumor Stroma. Experientia.

[B8] Jackson DG (1997). Human Leucocyte Heparan Sulphate Proteoglycans and Their Roles in Inflammation. Biochem Soc Trans.

[B9] Woude FJ van der, van Det NF (1997). Heparan Sulphate Proteoglycans and Diabetic Nephropathy. Exp Nephrol.

[B10] Vernier RL, Klein DJ, Sisson SP, Mahan JD, Oegema TR, Brown DM (1983). Heparan Sulfate – Rich Anionic Sites in the Human Glomerular Basement Membrane. Decreased Concentration in Congenital Nephrotic Syndrome. N Engl J Med.

[B11] Hoven MJ van den, Rops AL, Bakker MA, Aten J, Rutjes N, Roestenberg P, Goldschmeding R, Zcharia E, Vlodavsky I, vand V, Berden JH (2006). Increased Expression of Heparanase in Overt Diabetic Nephropathy. Kidney Int.

[B12] Gambaro G, Cavazzana AO, Luzi P, Piccoli A, Borsatti A, Crepaldi G, Marchi E, Venturini AP, Baggio B (1992). Glycosaminoglycans Prevent Morphological Renal Alterations and Albuminuria in Diabetic Rats. Kidney Int.

[B13] Deckert T, Feldt-Rasmussen B, Borch-Johnsen K, Jensen T, Kofoed-Enevoldsen A (1989). Albuminuria Reflects Widespread Vascular Damage. The Steno Hypothesis. Diabetologia.

[B14] Wasty F, Alavi MZ, Moore S (1993). Distribution of Glycosaminoglycans in the Intima of Human Aortas: Changes in Atherosclerosis and Diabetes Mellitus. Diabetologia.

[B15] Brown DM, Klein DJ, Michael AF, Oegema TR (1982). 35S-Glycosaminoglycan and 35S-Glycopeptide Metabolism by Diabetic Glomeruli and Aorta. Diabetes.

[B16] Kjellen L, Bielefeld D, Hook M (1983). Reduced Sulfation of Liver Heparan Sulfate in Experimentally Diabetic Rats. Diabetes.

[B17] Levy P, Picard J, Bruel A (1984). Evidence for Diabetes-Induced Alterations in the Sulfation of Heparin Sulfate Intestinal Epithelial Cells. Life Sci.

[B18] Han J, Woytowich AE, Mandal AK, Hiebert LM (2007). Heparanase Upregulation in High Glucose-Treated Endothelial Cells Is Prevented by Insulin and Heparin. Exp Biol Med (Maywood).

[B19] Katz A, Van Dijk DJ, Aingorn H, Erman A, Davies M, Darmon D, Hurvitz H, Vlodavsky I (2002). Involvement of Human Heparanase in the Pathogenesis of Diabetic Nephropathy. Isr Med Assoc J.

[B20] Han J, Mandal AK, Hiebert LM (2005). Endothelial Cell Injury by High Glucose and Heparanase Is Prevented by Insulin, Heparin and Basic Fibroblast Growth Factor. Cardiovasc Diabetol.

[B21] Gotlieb AI, Spector W (1981). Migration into an in Vitro Experimental Wound: a Comparison of Porcine Aortic Endothelial and Smooth Muscle Cells and the Effect of Culture Irradiation. Am J Pathol.

[B22] Jaques LB, Wice SM, Hiebert LM (1990). Determination of Absolute Amounts of Heparin and of Dextran Sulfate in Plasma in Microgram Quantities. J Lab Clin Med.

[B23] Kaji T, Yamada A, Miyajima S, Yamamoto C, Fujiwara Y, Wight TN, Kinsella MG (2000). Cell Density-Dependent Regulation of Proteoglycan Synthesis by Transforming Growth Factor-Beta(1) in Cultured Bovine Aortic Endothelial Cells. J Biol Chem.

[B24] Gharagozlian S, Borrebaek J, Henriksen T, Omsland TK, Shegarfi H, Kolset SO (2006). Effect of Hyperglycemic Condition on Proteoglycan Secretion in Cultured Human Endothelial Cells. Eur J Nutr.

[B25] Vogl-Willis CA, Edwards IJ (2004). High Glucose-Induced Alterations in Subendothelial Matrix Perlecan Leads to Increased Monocyte Binding. Arterioscler Thromb Vasc Biol.

[B26] Vogl-Willis CA, Edwards IJ (2004). High-Glucose-Induced Structural Changes in the Heparan Sulfate Proteoglycan, Perlecan, of Cultured Human Aortic Endothelial Cells. Biochim Biophys Acta.

[B27] Nugent MA, Iozzo RV (2000). Fibroblast Growth Factor-2. Int J Biochem Cell Biol.

[B28] Richardson JV, Wright CB, Hiratzka LF (1980). The Role of Endothelium in the Patency of Small Venous Substitutes. J Surg Res.

[B29] Colwell JA, Lopes-Virella MF (1988). A Review of the Development of Large-Vessel Disease in Diabetes Mellitus. Am J Med.

[B30] Tamsma JT, van den BJ, Bruijn JA, Assmann KJ, Weening JJ, Berden JH, Wieslander J, Schrama E, Hermans J, Veerkamp JH (1994). Expression of Glomerular Extracellular Matrix Components in Human Diabetic Nephropathy: Decrease of Heparan Sulphate in the Glomerular Basement Membrane. Diabetologia.

[B31] Ishihara M, Fedarko NS, Conrad HE (1987). Involvement of Phosphatidylinositol and Insulin in the Coordinate Regulation of Proteoheparan Sulfate Metabolism and Hepatocyte Growth. J Biol Chem.

[B32] Reizes O, Goldberger O, Smith AC, Xu Z, Bernfield M, Bickel PE (2006). Insulin Promotes Shedding of Syndecan Ectodomains From 3T3-L1 Adipocytes: a Proposed Mechanism for Stabilization of Extracellular Lipoprotein Lipase. Biochemistry.

[B33] Hsueh WA, Law RE (1999). Insulin Signaling in the Arterial Wall. Am J Cardiol.

[B34] Mather K, Anderson TJ, Verma S (2001). Insulin Action in the Vasculature: Physiology and Pathophysiology. J Vasc Res.

[B35] Zeng G, Nystrom FH, Ravichandran LV, Cong LN, Kirby M, Mostowski H, Quon MJ (2000). Roles for Insulin Receptor, PI3-Kinase, and Akt in Insulin-Signaling Pathways Related to Production of Nitric Oxide in Human Vascular Endothelial Cells. Circulation.

[B36] Olgemoller B, Schwaabe S, Gerbitz KD, Schleicher ED (1992). Elevated Glucose Decreases the Content of a Basement Membrane Associated Heparan Sulphate Proteoglycan in Proliferating Cultured Porcine Mesangial Cells. Diabetologia.

[B37] Kasinath BS (1995). Effect of Insulin on High-Glucose Medium-Induced Changes in Rat Glomerular Epithelial Cell Metabolism of Glycoconjugates. Arch Biochem Biophys.

[B38] Unger E, Kjellen L, Eriksson UJ (1991). Effect of Insulin on the Altered Production of Proteoglycans in Rib Cartilage of Experimentally Diabetic Rats. Arch Biochem Biophys.

[B39] Rohrbach DH, Wagner CW, Star VL, Martin GR, Brown KS, Yoon JW (1983). Reduced Synthesis of Basement Membrane Heparan Sulfate Proteoglycan in Streptozotocin-Induced Diabetic Mice. J Biol Chem.

[B40] Mandal AK, Puchalski JT, Lemley-Gillespie S, Taylor CA, Kohno M (2000). Effect of Insulin and Heparin on Glucose-Induced Vascular Damage in Cell Culture. Kidney Int.

[B41] Mandal AK, Lyden TW, Fazel A, Saklayen MG, Mehrotra B, Mehling B, Taylor CA, Yokokawa K, Colvin RV (1995). Heparin-Induced Endothelial Cell Cytoskeletal Reorganization: a Potential Mechanism for Vascular Relaxation. Kidney Int.

[B42] Yokokawa K, Kohno M, Mandal AK, Tahara H, Yanagisawa M, Takeda T, Kohne M (1994). Heparin Suppresses Endothelin-1 Peptide and MRNA Expression in Cultured Endothelial Cells of Spontaneously Hypertensive Rats. J Am Soc Nephrol.

[B43] Nader HB, Buonassisi V, Colburn P, Dietrich CP (1989). Heparin Stimulates the Synthesis and Modifies the Sulfation Pattern of Heparan Sulfate Proteoglycan From Endothelial Cells. J Cell Physiol.

[B44] Nader HB, Toma L, Pinhal MA, Buonassisi V, Colburn P, Dietrich CP (1991). Effect of Heparin and Dextran Sulfate on the Synthesis and Structure of Heparan Sulfate From Cultured Endothelial Cells. Semin Thromb Hemost.

